# Experiences of Rural and Urban Assisted Living Communities in Oregon During COVID-19 Pandemic

**DOI:** 10.1093/geroni/igab046.3594

**Published:** 2021-12-17

**Authors:** Ozcan Tunalilar, Jason Kyler-Yano, Sheryl Elliott, Sarah Dys, Paula Carder

**Affiliations:** 1 Portland State University, Portland, Oregon, United States; 2 Vital Research, LLC, Portland, Oregon, United States; 3 OHSU-PSU School of Public Health, Portland, Oregon, United States

## Abstract

This study presents findings on the impact of the COVID-19 pandemic as reported by a representative sample of Oregon assisted living communities (AL) between December 2020 and March 2021. Of the 559 AL eligible to participate, 346 completed eleven questions related to their experiences since March 2020. These questions covered topics such as access to personal protective equipment (PPE) and accurate information, communication with and support from government agencies, ability to find staff and new residents, ability to address pandemic-related concerns of residents’ families and staff, use of virtual visits and telehealth for residents, and visitor restrictions. Response categories ranged from 0 (strongly disagree) to 4 (strongly agree) and we coded “agree” and “strongly agree” responses as having experienced that issue. Among responding AL, 42% were located in rural or frontier areas. We present three findings. First, most AL experienced adverse impact due to COVID-19, especially regarding issues likely to be outside of their control compared to those within their control. Second, while almost all urban-based AL reported that their residents used virtual communication technologies and tools for telemedicine/telehealth (96%) or virtual social visits (96%), rural AL were less likely to report so (90% and 92%, respectively). Finally, rural AL experienced significantly greater staffing difficulties (75%) compared to their urban counterparts (82%). In sum, while all AL would benefit from better regulatory guidance on policies and access to PPE, rural AL might especially benefit from additional, context-specific resources.

